# Identification, characterization of two NADPH-dependent erythrose reductases in the yeast *Yarrowia lipolytica* and improvement of erythritol productivity using metabolic engineering

**DOI:** 10.1186/s12934-018-0982-z

**Published:** 2018-08-29

**Authors:** Huiling Cheng, Siqi Wang, Muhammad Bilal, Xuemei Ge, Can Zhang, Patrick Fickers, Hairong Cheng

**Affiliations:** 1 State Key Laboratory of Microbial Metabolism, and School of Life Sciences & Biotechnology, Shanghai Jiao Tong University, Shanghai, China; 2 College of Light Industry and Food Engineering, Nanjing Forestry University, Nanjing, China; 3 School of Pharmacy, Shanghai Jiao Tong University, Shanghai, China; 4 Microbial Processes and Interactions, TERRA Teaching and Research Centre, University of Liège-Gembloux Agro-Bio Tech, Gembloux, Belgium

**Keywords:** Erythrose reductase, Erythritol, *Yarrowia lipolytica*, NADPH, Metabolic engineering

## Abstract

**Background:**

Erythritol is a four-carbon sugar alcohol with sweetening properties that is used by the agro-food industry as a food additive. In the yeast *Yarrowia lipolytica*, the last step of erythritol synthesis involves the reduction of erythrose by specific erythrose reductase(s). In the earlier report, an erythrose reductase gene (*YALI0F18590g*) from erythritol-producing yeast *Y. lipolytica* MK1 was identified (Janek et al. in Microb Cell Fact 16:118, 2017). However, deletion of the gene in *Y. lipolytica* MK1 only resulted in some lower erythritol production but the erythritol synthesis process was still maintained, indicating that other erythrose reductase gene(s) might exist in the genome of *Y. lipolytica*.

**Results:**

In this study, we have isolated genes g141.t1 (*YALI0D07634g*) and g3023.t1 (*YALI0C13508g*) encoding two novel erythrose reductases (ER). The biochemical characterization of the purified enzymes showed that they have a strong affinity for erythrose. Deletion of the two ER genes plus g801.t1 (*YALI0F18590g*) did not prevent erythritol synthesis, suggesting that other ER or ER-like enzymes remain to be discovered in this yeast. Overexpression of the newly isolated two genes (ER10 or ER25) led to an average 14.7% higher erythritol yield and 31.2% higher productivity compared to the wild-type strain. Finally, engineering NADPH cofactor metabolism by overexpression of genes *ZWF1* and *GND1* encoding glucose-6-phosphate dehydrogenase and 6-phosphogluconate dehydrogenase, respectively, allowed a 23.5% higher erythritol yield and 50% higher productivity compared to the wild-type strain. The best of our constructed strains produced an erythritol titer of 190 g/L in baffled flasks using glucose as main carbon source.

**Conclusions:**

Our results highlight that in the *Y. lipolytica* genome several genes encode enzymes able to reduce erythrose into erythritol. The catalytic properties of these enzymes and their cofactor dependency are different from that of already known erythrose reductase of *Y. lipolytica*. Constitutive expression of the newly isolated genes and engineering of NADPH cofactor metabolism led to an increase in erythritol titer. Development of fermentation strategies will allow further improvement of this productivity in the future.

**Electronic supplementary material:**

The online version of this article (10.1186/s12934-018-0982-z) contains supplementary material, which is available to authorized users.

## Background

Erythritol (1,2,3,4-butanetetrol) is a four-carbon sugar alcohol with sweetening properties and applications in the agro-food and pharmaceutical industries [1–3]. It is most commonly produced by yeast, namely *Torula corallina* [4, 5], *Candida magnoliae* [6, 7], *Pseudozyma tsukubaensis* [8], and *Trichosporonoides megachiliensis* [9] with *Y*_*P/S*_ conversion yield ranging from 0.43 to 0.61 g/g. In yeast, erythritol is synthesized via the pentose phosphate pathway (PPP, [10, 11]) as an osmoprotectant in response to osmotic stress [1].

Recently, the yeast *Yarrowia lipolytica* has also been found to be an efficient erythritol producer [2]. Several processes based on wild-type strains have been developed [12–14], but the most promising processes are based on metabolically engineered strains. Overexpression of genes involved in PPP, namely transketolase (*TKL1*, YALI0E06479g), transaldolase (*TAL1*, YALI0F15587g) and erythrose reductase (*ylER*, *YALI0F18590g*), allowed the erythritol productivity to increase with various magnitudes (from 16 to 200%) [11, 15, 16]. Other engineering strategies based on improved carbon source metabolism [10, 16] or co-factor engineering [11] have also been employed with success to improve erythritol production.

The final step of erythritol synthesis is the reduction of erythrose from the PPP by erythrose reductase (ER) with concurrent NAD(P)H oxidation [7, 17]. Several studies have been conducted on ER from the yeast. One ER has been purified and characterized from *Candida magnoliae* [7], while three ER isozymes were found in *Trichosporonoides megachiliensis* (ER-I, ER-II and ER-III isozymes [18], and two in *Moniliella* sp. (MsER1 and MsER2 isozymes, [19]). However, the exact biological activity and properties of these isozymes in erythrose reduction remain to be characterized. Recently, gene *YALI0F18590g* was reported as encoding an ER in *Y. lipolytica* [15]. Overexpression of the latter in strain *Y. lipolytica* MK1 yielded an erythritol titer of 44.4 g/L and productivity of 0.77 g/L h.

In order to get more insights on ER in *Y. lipolytica* and with the aim to further increase the erythritol production, we identified and characterized two additional ER (namely ER10 and ER27) in *Y. lipolytica* strain CGMCC7326, a strain able to produce erythritol with very high titer (more than 150 g/L, [14, 20]). Overexpression of those ER encoding genes together with *ZWF1* (*YALI0E22649g*) and *GND1* (*YALI0B15598g*) that allow NADPH to be replenished, yielded significantly improved erythritol productivity.

## Results and discussion

### Identification of erythrose reductase-encoding genes in *Y. lipolytica* CGMCC7326

In erythritol producing yeast, the final step of erythritol synthesis consists in the reduction of erythrose by specific erythrose reductase [10, 11]. Recently, Janek et al. [15] identified an ylER enzyme (*YALI0F18590p*) in *Y. lipolytica* based on sequence similarity with erythrose reductase from *Candida magnoliae* [7]. These ER enzymes, belonging to the aldose reductase family (ALR), have also been reported mainly dependent on NADPH as a redox co-factor [5, 18, 21, 22]. Different ER isozymes have been reported in *T. megachiliensis* [18] and *Moniliella* sp. [19], and these erythrose reductases are strictly dependent on NADPH as a redox co-factor [18, 21, 22]. We searched the gene function from the genome annotation of *Y. lipolytica* strain CGMCC 7326 for enzymes with reductase and NADPH as keywords. Then only enzymes with NADPH dependent reductase were obtained from the genome annotation.

**Fig. 1:**
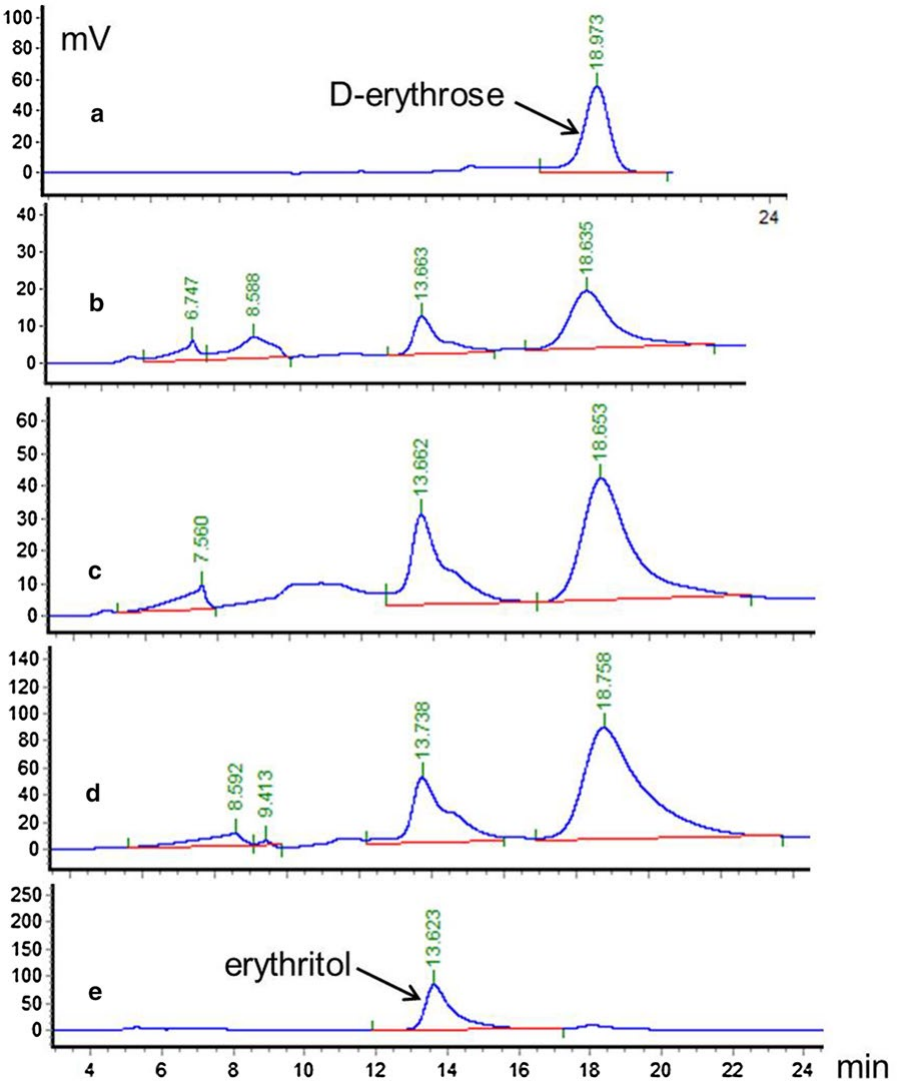
HPLC analysis of reaction product catalyzed by the crude extracts of *E. coli* overexpressing putative ER enzymes. **a** standard of d-erythrose; **b**–**d** reaction product obtained with a cell extract of *E. coli* strain HCE102, HCE110, and HCE111, respectively; **e** standard of erythritol

This led to the identification of 12 putative genes, including *g141.t1* (*ER10*), *g3023.t1* (*ER25*) and *g801.t1* (*ER27*) (Table 1). In order to confirm the predicted catalytic activity, these twelve genes were PCR amplified from the genomic DNA of strain CGMCC7326 using primers listed in Additional file 1: Table S1, cloned into a pET28a vector and expressed in *E. coli* BL21(DE3). Assay for reductase activity on cell extract of IPTG-induced cells using d-erythrose as a substrate and NADPH as a co-factor led to the identification of strain HCE102 (gene *g141.t1*, *ER10*), HCE110 (gene *g3023.t1*, *ER25*) and HCE111 (gene *g801.t1*, *ER27*) with NADPH-dependent erythrose reductase activity. Indeed, reduction of d-erythrose into erythritol could be detected with the above-mentioned cell extract as shown in Fig. 1. No reductase activity could be found in the crude extract of non-transformed *E. coli* in those experimental conditions.

**Table 1:** Putative NADPH-dependent reductases identified in the *Y. lipolytica* CGMCC7326 genome

Gene ID in *Y. lipolytica* CGMCC7326	Counterpart gene ID in *Y. lipolytica* CLIB122	Protein	Predicted function
g141.t1	YALI0D07634g	ER10^a^	Probable NAD(P)H-dependent d-Xylose reductase xyl1
g413.t1	YALI0F09075g	ER05	NADPH-dependent aldehyde reductase ARI1
g414.t1	YALI0F09097g	ER08	Putative NADPH-dependent methylglyoxal reductase GRP2
g801.t1	YALI0F18590g	ER27^a^	putative NADP-dependent aldo/keto reductase
g973.t1	YALI0A15906g	ER17	putative NADPH-dependent galactose-induced protein of aldo–keto reductase
g3023.t1	YALI0C13508g	ER25^a^	Putative NADPH-dependent aldo–keto reductase gene
g3251.t1	YALI0C20251g	ER20	Putative NADPH-dependent methylglyoxal reductase GRP2
g3449.t1	YALI0C06171g	ER22	NADP(H)-dependent oxidoreductase YfmJ
g3584.t1	YALI0C02805g	ER14	probable NADPH-dependent beta-ketoacyl reductase
g5171.t1	YALI0B15268g	ER18	NADPH-dependent alpha-ketoamide reductase
g5456.t1	YALI0B01298g	ER24	NADP-preferring aldehyde dehydrogenase
g5767.t1	YALI0B07117g	ER16	probable NADP(H)-dependent aldo–keto reductase gene

^a^Indicates that the protein has d-erythrose reductase activity

**Fig. 2:**
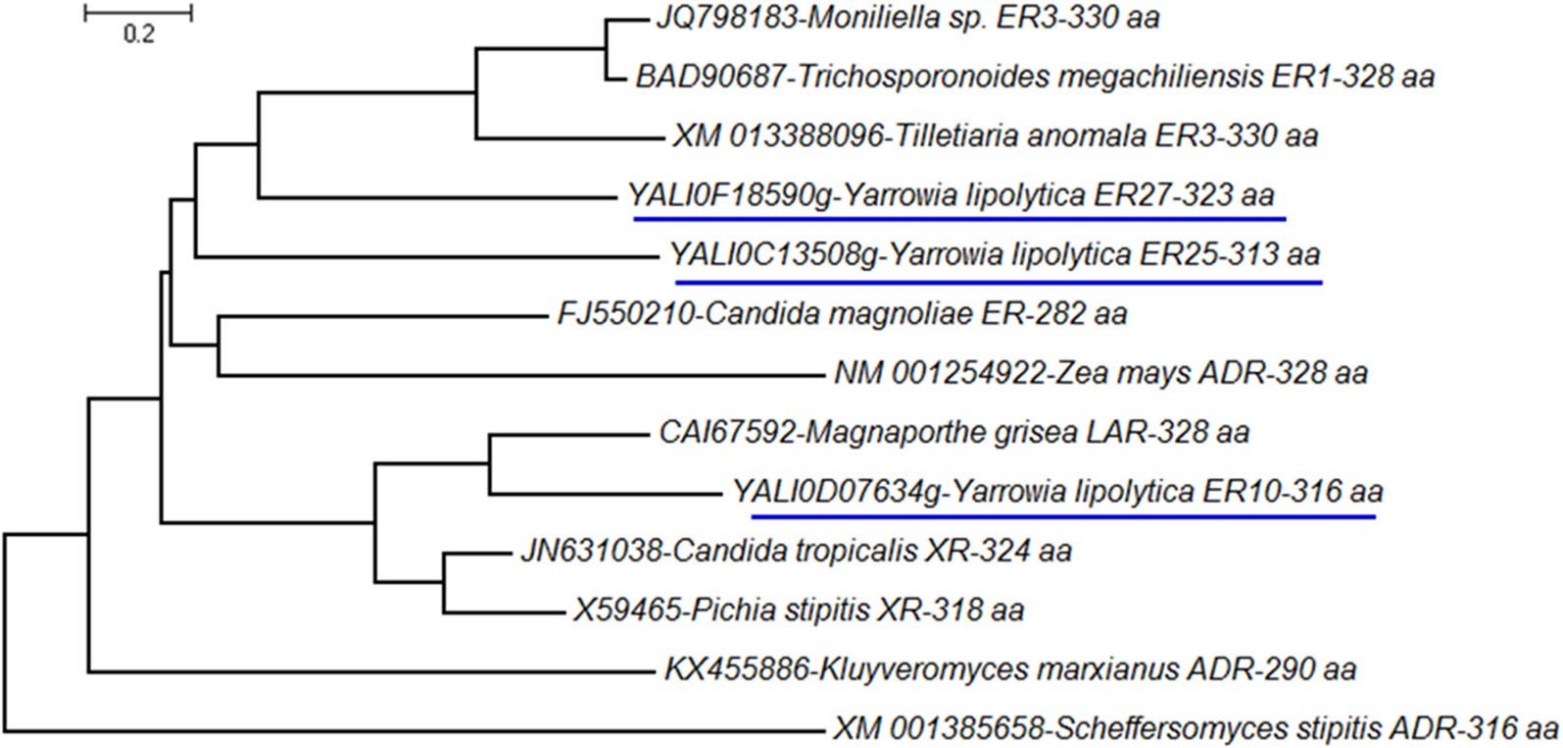
Phylogenetic analysis of the three erythrose reductases (ER10, ER25, and ER27, underlined in blue) from *Y. lipolytica* and other selected reductases (*ADR* aldose reductase, *LAR* l-arabinose reductase, *XR* xylose reductase). The phylogenetic tree was constructed based on the alignment of full amino acid sequences. All the analyzed sequences of aldose reductase enzymes were retrieved from GenBank and SWISS-PROT databases

Protein BLAST search using a translated sequence of genes *g141.t1*, *g3023.t1*, and *g801.t1* as a query, highlighted in *Y. lipolytica* strain CLIB122 genes *YALI0D07634g*, *YALI0C13508g*, and *YALI0F18590g*, respectively, with 100% identity. Therefore, gene *g801.t1* corresponds to the gene *YALI0F18590g* identified as encoding *yl*ER in *Y. lipolytica* strain MK1 [15]. Amino acid sequence identity between ER10, ER25, and ER27 ranged from 26.8 to 31.8%, while identity ranged from 24.0 to 38.0% for *C. magnoliae* ER (ACT78580.1), *T. megachiliensis* ER1 (BAD90687), *Tilletiaria anomala* ER3 (XP_013243550.1) and *Moniliella* sp. ER3 (AGB07593.1) (Additional file 1: Table S2). To verify Blast analysis, a phylogenetic tree was also constructed with a full-length amino acid sequence of ER10, ER25, ER27, other selected ER and NADPH-dependent aldose reductases using the MEGA7 software. The most striking point shown in Fig. 2 is that *Y. lipolytica* ER10 is closely related to l-arabinose reductase from the fungus *Magnaporthe grisea* (54.27% identity) and is relatively distant from *Y. lipolytica* ER25 and ER27 (26.8% and 31% of identity, respectively). This in silico analysis suggests that ER10 has evolved differently from ER25 and ER27 in the ALR family.

### Disruption of three ERs gene and its effects on erythritol synthesis

To confirm that the three putative identified ERs are indeed involved in erythritol biosynthesis, the corresponding encoding gene (i.e. *ER10*, *ER25* and *ER27*), were disrupted in *Y. lipolytica* strain CGMCC7326. Disruption cassettes, namely *pINA*-*UP*_*ER10*_-*DW*_*ER10*_, *pINA*-*UP*_*ER25*_-*DW*_*ER25*_, *pINA*-*UP*_*ER27*_-*DW*_*ER27*_, were constructed as described in “Methods”, and used to disrupt the corresponding gene separately or simultaneously. The disrupted mutants were grown in EPMG medium and erythritol was quantified directly after glucose depletion in the medium. As shown in Table 2, the specific growth rate of the disrupted mutants were significantly decreased from that of the parental strain. Compared to the parental strain CGMCC7326, disruption of genes g3023.t1 (*ER25*, strain HCY101) and g801.t1 (*ER27*, strain HCY102) resulted in a lower erythritol production (11.1% and 17.1% decrease, respectively), a lower productivity (30% and 39% decrease, respectively) and a lower yield (11.7% and 17.6% decrease, respectively) (Table 2). Unexpectedly, the disruption of gene g141.t1 (*ER10*, strain HCY100) led to an increase in erythritol titer and yield (10% and 9%, respectively). Disruption of the three ER encoding genes in strain HCY103 led to a lower erythritol production (decreased by 36.8%), a lower productivity (decreased by 61.2%) and a lower yield (decreased by 37.2%). From this, it could be concluded that the three identified genes encode proteins involved in the erythritol biosynthetic pathway in *Y. lipolytica* CGMCC7326, deletion of *ER10* gene increased erythritol biosynthetic capacity, while deletion of *ER25* and *ER27* gene decreased erythritol biosynthetic capacity. However, the ability of the multiple disrupted strain HCY103 to synthesize erythritol highlights that alternative biochemical pathway for erythritol synthesis or other unknown *ER* gene(s) remain to be identified.

**Table 2:** Erythritol production for strain CGMCC7326 and *ER* gene disrupted strains HCY100, HCY101, HCY102, HCY103

*Y. lipolytica* strains	Erythritol production (g/L)	Q_ERY_ (g/L h)	Y_ERY_ (g/g)	Time (h)	Specific growth rate (h^−1^)
CGMCC7326	152 ± 4	1.55	0.51	98	0.042
HCY100	168 ± 6	1.50	0.56	112	0.034
HCY101	135 ± 5	1.09	0.45	124	0.035
HCY102	126 ± 6	0.95	0.42	132	0.034
HCY103	96 ± 5	0.60	0.32	160	0.029

Erythritol was quantified directly after glucose exhaustion in the culture medium. The values provided are the means of three independent replicates

### Purification of erythrose reductases

To further characterize the catalytic activity of ER10, ER25, and ER27, the enzymes were separately produced using *E. coli* and purified. After 10 h of induction of the corresponding *E. coli* BL21(DE3) strains, namely HCE102, HCE110 and HCE111, cells were collected and proteins were purified by Ni–NTA and gel filtration chromatography (see “Methods” for details). The apparent molecular weights of ER10, ER25 and ER27 deduced from SDS-PAGE corresponded to the one calculated from the amino acid sequence (35.8 kDa, 35.0 kDa and 36.2 kDa, respectively; Additional file 1: Figure S1). The purity of the three purified ER, estimated by scanning densitometry of the SDS-PAGE gel after protein staining with Coomassie brilliant blue G250 was higher than 98% for ER10, ER25 and ER27.

### Determination of the kinetic parameters of erythrose reductases

The catalytic constants were deduced from the non-linear regression curves (Table 3 and Additional file 1: Figure S2). All three of the enzymes were found able to convert erythrose into erythritol. ER25 showed the highest values of *K*_*cat*_ (19.7 s^−1^) and *K*_*cat*_*/K*_*m*_ (1.09 mM^−1^ s^−1^) demonstrating a higher ability to catalyze the reduction of erythrose into erythritol than ER10 (11.43 s^−1^ and 0.98 mM^−1^ s^−1^, respectively) and ER27 (15.8 s^−1^ and 0.61 mM^−1^ s^−1^) respectively.

**Table 3:** Kinetic parameters of erythrose reductase encoded by gene *g141.t1* (*ER10*), *g3023.t1* (*ER25*) and *g801.t1* (*ER27*)

Enzymes	Substrate/cofactor	*K*_*m*_ (mM)	*V*_*max*_ (*μ*mol/min)	*K*_*cat*_ (s^−1^)	*K*_*cat*_/*K*_*m*_ (mM^−1^s^−1^)
ER10	d-Erythrose/NADPH	11.46	8.17	11.43	0.98
ER10	Erythritol/NADP^+^	159.7	0.62	0.86	0.005
ER25	d-Erythrose/NADPH	18.06	14.58	19.70	1.09
ER27	d-Erythrose/NADPH	25.99	12.01	15.8	0.61

Cofactors (NADPH or NADP^+^) concentrations were 2 mM. *K*_*m*_ is for d-erythrose or erythritol

**Fig. 3:**
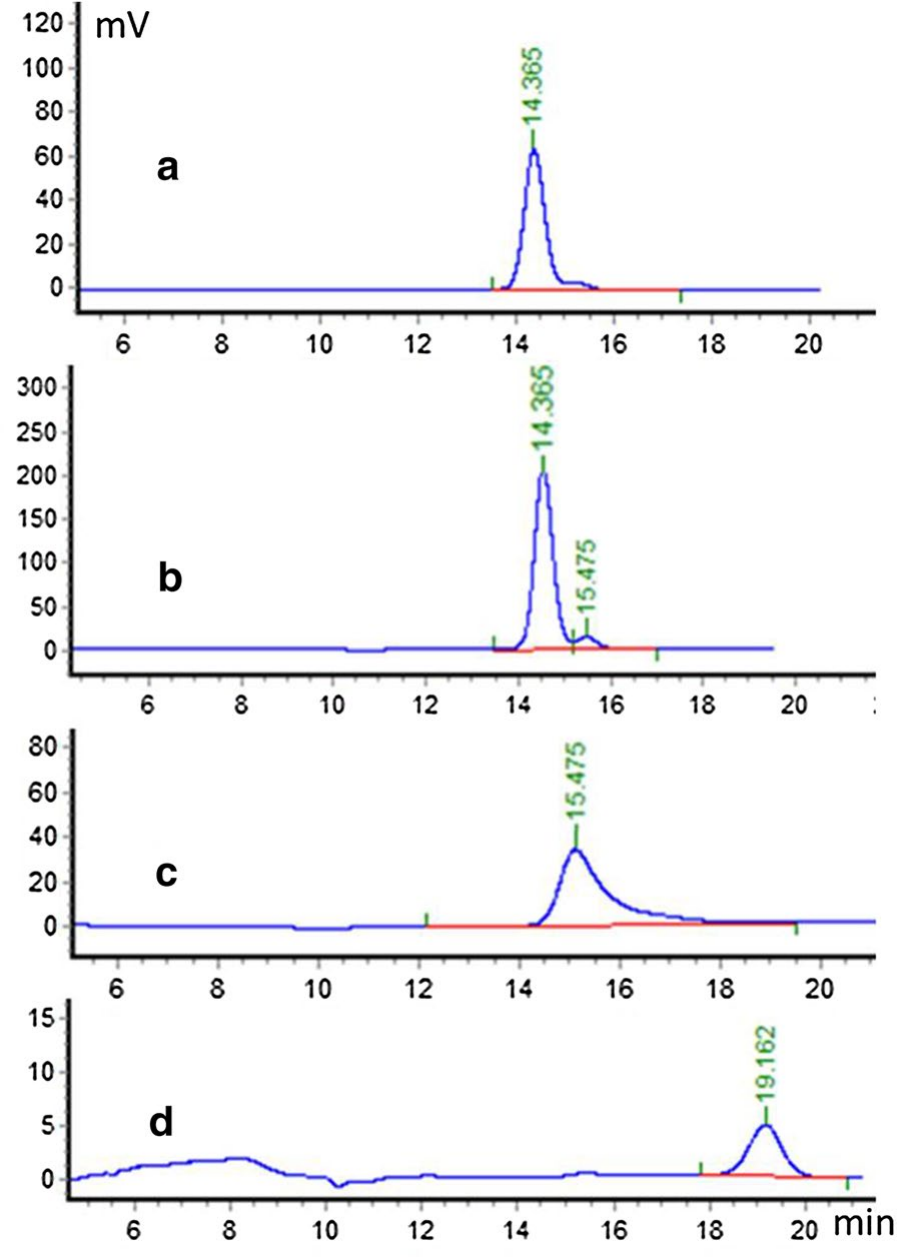
HPLC analysis of the reaction product of ER10 reductase in the presence of erythritol and NADP^+^. **a** erythritol standard; **b** reaction mixture containing erythritol, NADP^+^, and ER10; **c** erythrulose standard; **d** erythrose standard

We also assessed the ability of the purified ER to catalyze the backward reaction (i.e. the oxidation of erythritol) at pH8.0. For this purpose, erythritol was used as a substrate at different concentrations (from 20 to 1500 mM) and NADP^+^ was used as the cofactor (2 mM). As shown in Table 3, ER10 was found able to oxidize erythritol in the presence of NADP^+^, however with a lower efficiency than for the forward reaction (0.86 s^−1^ and 0.005 mM^−1^ s^−1^, respectively). Surprisingly, the reaction product, analyzed by high-performance liquid chromatography, was found to correspond to erythrulose and not to erythrose (Fig. 3). It has been reported recently in *Y. lipolytica*, that erythrulose, the first intermediate of erythritol catabolism, is obtained from an oxidation reaction catalyzed by an erythritol dehydrogenase, namely EYD1 (gene *YALI0F01650g*, [23]). However, such an oxidative activity on erythritol has been also reported for the ER from *C. magnoliae* KFCC11023 but only at a low rate and in alkaline conditions [7]. Based on kinetic parameters, the reaction catalyzed by ER10 in cells is more likely the reduction of erythrose to erythritol than its oxidation of erythritol to l-erythrulose. As described in the above section, the erythritol titer of strain HCY100 increased by 10% than that of the wild-type strain CGMCC7326, when *ER10* gene was disrupted (Table 2). The utilization of erythritol by *Y. lipolytica* slowed down when *ER10* gene was disrupted, and erythritol synthesis can be compensated by ER25, ER27 or other unidentified aldose reductases with erythrose reductase activity. For ER25 and ER 27, the oxidation of erythritol could not be detected in the experimental conditions tested (data not shown).

### Biochemical properties of purified ER

**Fig. 4:**
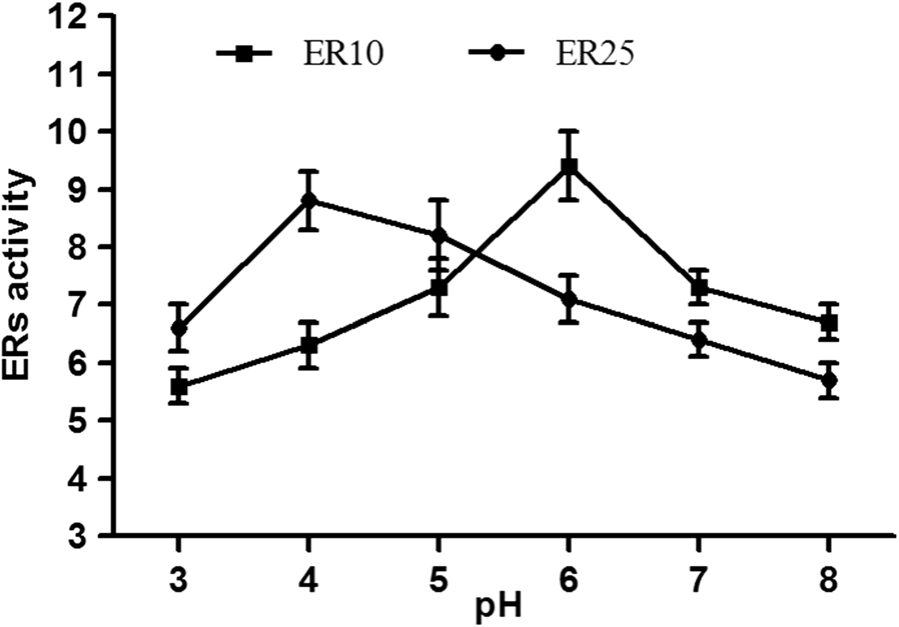
Effect of pH on the specific activity of ER10 and ER25 reductases. Purified enzymes were mixed with Mcilvaine’s buffer and assayed at various pH values ranging from 3.0 to 8.0. The values provided are the means of three independent replicates; the standard deviations represented less than 10% of the means

In *Y. lipolytica* strain MK1, the erythrose reductase encoded by gene *YALI0F18590g* (namely *yl*ER) displays the highest activity at pH 3 in the presence of 0.25 mM of Zn^2+^ [15]. Similarly, the effect of pH and divalent metal ions on the catalytic activity was determined for ER10 and ER25. The erythrose reductase activity was tested at pH values ranging from 3 to 8. As shown in Fig. 4, the optimal pH for ER10 reductase activity is 6.0; the activity was reduced by 10% at pH 5.0 and 7.0. At pH 3.0 and 8.0, the enzymatic activity was reduced by 37% and 32%, respectively. The optimal pH for ER25 reductase activity is 4, and activity significantly decreases at pH 3 and 8 (26% and 40%, respectively). The optimal pH value of ER10 (pH 6.0) and ER25 (pH 4.0) are somewhat higher than the one reported for *yl*ER (pH 3.0) by Janek et al. [15]. These results showed that the three ERs characterized so far in *Y. lipolytica* prefer acidic conditions for maximal activity, which is a typical characteristic of aldo–ketoreducatse (AKR, [24]).

Similarly, to *yl*ER from *Y. lipolytica* strain MK1, the addition of Zn^2+^ increases the activity of both ER10 and ER25 (Additional file 1: Table S3). Ni^2+^ also seems to enhance ER25 activity at concentrations ranging from 0.5 to 1 mM but reduced significantly the activity of ER10. Other divalent ions, such as Cu^2+^, Mn^2+^, or Ca^2+^ also inhibit the activity of both enzymes (Additional file 1: Table S3).

### Substrate and cofactor specificity of ER10 and ER25

The *yl*ER from *Y. lipolytica* MK1 was also shown to have a high specific activity for d-erythrose as well as for d-arabinose, d-fructose, d-galactose, and d-glucose [15]. Similar experiments showed that ER10 and ER25 present the highest specific activity for d-erythrose (8.2 and 14.6 U/mg, respectively; Table 4). In contrast to ER25, ER10 is able to use l-arabinose, d-xylose, d-ribose, and d-xylulose as substrate, however with a lower specificity (4.3, 2.4, 3.4, 1.3 U/mg, respectively; Table 4). Both ER10 and ER25 were found unable to reduce d-fructose, d-galactose and d-glucose in contrast to the *yl*ER from strain MK1 (data not shown). The substrate specificity of ER10 was consistent with the erythrose reductase from *C. magnoliae*, which also has enzymatic activity against d-erythrose, d-xylose, and d-ribose [21].

**Table 4:** Substrates specificity of ER10 and ER25 enzymes

Substrates	ER10^a^	Reductase activity (U/mg protein)		
		ER10^b^	ER25^a^	ER25^b^
d-Erythrose	8.2 (100)	8.3 (100)	14.6 (100)	14.1 (100)
l-Arabinose	4.3 (52.4)	4.1 (49.4)	1.8 (12.3)	1.5 (10.6)
d-Xylose	2.4 (29.3)	2.5 (30.1)	ND	ND
d-Ribose	3.4 (41.5)	3.1 (37.3)	ND	ND
d-Xylulose	1.3 (15.9)	1.1 (13.3)	ND	ND

*ND* no activity detected. (Relative activity compared to d-erythrose). The values provided are the means of three independent replicates; the standard deviations represented less than 10% of the means

^a^Activity determined in 50 mM phosphate buffer at its optimal pH (pH 6 for ER10, and pH 4 for ER25)

^b^Activity determined in 50 mM phosphate buffer with high osmotic condition (200 g/L glucose) at its optimal pH

Yeast ALRs can be classified into two groups according to their strict dependency to NADPH or their dual coenzyme (i.e. NADPH and NADH) specificity [25, 26]. Janek et al. [15] has already demonstrated that *yl*ER is dependent on NADH as a co-factor. To investigate the cofactor preference of ER10 and ER25, reduction of d-erythrose was measured in the presence of both NADPH and NADH. Reduction of erythrose was only detected when NADPH was used as a cofactor (8.2 U/mg protein for ER10, and 14.6 U/mg protein for ER25). Erythrose reduction could not be measured in the presence of NADH even at high concentration (10 mM). Similarly, oxidation of erythritol by ER10 could only be detected in the presence of NADP (0.65 U/mg of protein). The specific motif IPKSXXXR has been reported as a common catalytic feature found in both aldo–keto reductase (AKR) and short-chain dehydrogenase/reductase (SDR) implicated in cofactor binding. Indeed, it has been demonstrated that the lysine residue binds the 2′-phosphate of NADPH [27, 28]. Protein sequence analysis highlighted such a motif for ER25 at position 266 (i.e. IPKSNNVER) and for ER10 at position 258 despite the serine residue is replaced by a threonine (i.e. IPKSTRSVER) (Additional file 1: Figure S3). In contrast, this motif could not be detected in ER27. This confirms that both ER10 and ER25 are NADPH dependent reductase.

### Overexpression of erythrose reductase

With the goal to increase erythritol productivity, gene encoding ER10 (*g141.t1*), ER25 (*g3023.t1*) and ER27 (*g801.t1*) were overexpressed under the control of the strong constitutive promoter hp4d, either separately or simultaneously, in the *Y. lipolytica* strain CGMCC7326. Gene overexpression was confirmed by qPCR (12-fold increased expression in average as compared to the wild-type strain, data not shown), demonstrating that the expression cassettes were functional in transformed strains. The engineered strains were grown in EPMG medium until the carbon source (glucose 300 g/L) was totally consumed. As shown in Table 5, the specific growth rates of the ER overproducing strains are not significantly different of that of the parental strain. Strains HCY104 (php4d-ER10), HCY105 (php4d-ER25) and HCY106 (php4d-ER27) all exhibited an increased erythritol titer as compared to the parental strain CGMCC7326 (12%, 13% and 16%, respectively; Table 5). Engineered strain AJD pAD-ylER overexpressed *YALI0F18590g* (*ER27* gene in this study) in the starting strain *Y. lipolytica* MK1 resulted in an enhanced erythritol production of 44.44 g/L, 20% higher than that of the control strain *Y. lipolytica* MK1, and 4% higher than the strain HCY106 (13% higher than that of the control strain CGMCC7326). However, the productivity of the HCY106 strain was higher relative to the engineered strain AJD pAD-ylER (2.2 g/L h vs 0.77 g/L h). The time for glucose consumption was also reduced by 13% for strain overexpressing ERs encoding gene as compared to the parental strain. Overexpressing strains also exhibited a slightly higher glucose uptake rate (Table 5). Glucose was totally depleted after 96 h for the control strain, while only 84 h were needed for all engineered strains.

**Table 5:** Erythritol production during culture of strains

Strains	Erythritol production (g/L)	Q_ERY_ (g/L h)	Y_ERY_ (g/g)	T (h)	r_GLU_ (g/h L)	Specify growth rate (h^−1^)
*Y. lipolytica* CGMCC7326	154 ± 9.5	1.6	0.51	96	3.12	0.043
HCY104 (*php4d*-*ER10*)	174 ± 8.5	2.1	0.58	84	3.57	0.047
H CY105 (*php4d*-*ER25*)	177 ± 8.5	2.1	0.59	84	3.57	0.046
HCY106 (*php4d*-*ER27*)	182 ± 7.5	2.2	0.61	84	3.57	0.045
HCY107 (*php4d*-*ER10*-*25*-*27*)	178 ± 7.2	2.1	0.59	84	3.57	0.046
HCY108 (*php4d*-*ER10*-*25*-*27*, *php8d*-*ZWF1*-*GND1)*	190 ± 7.5	2.4	0.63	80	3.75	0.045

Cultures were performed in 2-L baffled flasks. t: the fermentation time when glucose was completely consumed. The values provided are the means of three independent replicates; the standard deviations represented less than 5% of the means

Next, we overexpressed the gene coding for ER10, ER25, and ER27 simultaneously in strain CGMCC7326 with the aim to further increase the erythritol productivity. In the resulting HCY107 strain, expression of genes *YALI0D07634g* (*ER10*), *YALI0C13508g* (*ER25*), and *YALI0F18590g* (*ER27*) were increased by 5.3, 15.7 and 16.4-fold, respectively, as compared to the parental strain (data not shown). However in contrast to our expectation, erythritol productivity and yield for strain HCY107 were not further increased as compared to that of strain HCY104, HCY105, and HCY106, respectively (Table 5). To explain this observation, we hypothesized that the intracellular pool of NADPH was not sufficient to sustain the ER activity in strain HCY107.

### Engineering of cofactor metabolism

As demonstrated above, the reduction of erythrose by ER needs NADPH as a cofactor. Therefore, regenerating this cofactor from NADP is a key factor to increase erythritol productivity in strain HCY107. The first reaction of the PPP pathway, catalyzed by the glucose-6P dehydrogenase (G6PD), generates NADPH from glucose-6P and NADP^+^. In yeast, it has been suggested that G6PD has a major role in NADPH production [29]. The 6-phosphogluconate dehydrogenase, that catalyzes the third reaction of the PPP pathway, also generates NADPH using 6-phosphogluconate as substrate. In *Y. lipolytica*, these two enzymes are encoded by gene *ZWF1* (*YALI0E22649g*) and *GND1* (*YALI0B15598g*), respectively [11]. Therefore, overexpressing these two genes in strain HCY107 will regenerate the NADPH consumed by ER. Moreover, the flux of carbon (glucose-6P from glycolysis) will be redirected toward the PPP pathway, and thus the synthesis of erythrose, the substrate of ER. In the resulting strain HCY108 (*php4d*-*ER10*-25-27-pTEF-*ZWF1*-*GND1*), erythritol yield and productivity were further improved by 6.7% and 14.3%, respectively, as compared to strain HCY107, and 23.5% higher erythritol yield and 50% higher productivity compared to the wild-type strain CGMCC7326 (Table 5). Though the erythritol titer was only average 6–7% higher for the strain HCY108 (overexpression of the three *ER* genes plus *zwf1* and *gnd1* genes) than that of HCY107 (overexpression of the three *ER* genes), the average 6–7% improvement was also important to increase its economy. The titer could be further improved by overexpression of other key genes related to erythritol synthesis such as transketolase or ribose 5-phosphate epimerase genes. This demonstrates that overexpression of genes *ZWF1* and *GND1* have some positive impact on erythritol synthesis.

In contrast to other reports [11], the byproducts such as mannitol and arabitol were not increased in all engineered strains, as compared to the wild-type, while erythritol production increased significantly for all engineered strains (Additional file 1: Figure S4). The mannitol and arabitol dehydrogenase activities which are responsible for byproducts mannitol and arabitol synthesis are very low, 0.021 U/ml and 0.015 U/mL, almost the same to the wild type. In contrast to the control strain CGMCC7326, the engineered strain HCY108 produced less biomass and citrate (6.3 g/L citrate for HCY108 and 13.4 g/L for the control strain CGMCC7326), when fermented at starting pH 6.5, resulting in higher pH than the control strain CGMCC7326 (Additional file 1: Figure S5). The results indicated that overexpression of the three ER genes plus *ZWF1* and *GND1* could push glucose metabolism into phosphate pentose pathway leading to synthesize more erythritol (Table 5).

## Conclusions

We have isolated, purified and characterized two novel ER enzymes of *Y. lipolytica*. They were found able to reduce erythrose to erythritol specifically in the presence of NADPH as a cofactor. In contrast to previously reported ER in the yeast *Y. lipolytica*, ER10 and ER25 have an optimal catalytic activity at higher pH than that of YALI0F18590p (ER27 in this study). Overexpression of genes encoding these ER together with the engineering of NADPH metabolism allowed significantly increased erythritol production titers.

## Methods

### Chemicals

d-erythrose, l-erythrulose, d-erythritol, d-arabitol, l-arabinose, d-xylose, d-ribose, and d-xylulose were obtained from Sigma-Aldrich (St. Louis, United States). Coomassie brilliant blue R-250, cofactor NADP, NAD, NADH, NADPH, and antibiotics were purchased from Sangon Biotech (Shanghai, China). The chromatographic media were procured from GE Healthcare Life Sciences (Sweden). All other chemicals were of analytical grade and used as such without any purification.

### Strains, media, and culture conditions

The *Escherichia coli* and *Y. lipolytica* strains used in this study are listed in Additional file 1: Table S1. The *E. coli* strains were grown at 37 °C in Luria–Bertani medium supplemented with kanamycin sulfate (50 mg/L). The *Y. lipolytica* strains were grown at 28 °C in YPD (10 g/L yeast extrac, 5 g/L tryptone, and 10 g/L dextrose) or YNB medium (10 g/L yeast nitrogen base without amino acids, 5 g/L ammonia sulfate) supplemented sucrose (20 g/L, YNBS), lactose (20 g/L, YNBL) or xylitol (20 g/L, YNBX). Hygromycin (200 μg/mL) was added in YPD when necessary to screen transformants. For erythritol synthesis, the EPMG medium was employed (per L): 300 g glucose, 8 g yeast extract, 8 g corn syrup, 2 g ammonium citrate, 0.05 g MnSO_4_·H_2_O, 0.05 g ZnSO_4_·7H_2_O and 0.01 g CuSO_4_·5H_2_O, initial pH 6.5. For solid media, agar (15 g/L) was added. Shake-flask cultures for erythritol production were performed in triplicate using 2 L baffled flasks containing 500 mL EPMG medium, at 30 °C and 200 rpm. Cultures were performed until glucose was depleted. All the baffled Erlenmeyer flasks containing culture medium were weighed both at the start of fermentation and then prior to taking samples aliquots for analysis. Sterile water was supplemented to compensate for the medium evaporation during fermentation. At the time of sampling, the resulting reduced weight was replenished by the addition of same weight of distilled water. At the end of the fermentation, the final weight was essentially the same as the initial weight.

### Analytical methods and calculation of erythritol production parameters

Erythritol, erythrose, l-erythrulose, mannitol, arabitol, arabinose, arabitol, xylose, xylitol, ribose, ribitol, and glucose were quantified by HPLC using a refractive index detector (Shodex RI101) and a Shodex SP0810 ion exclusion column (300 × 8 mm). Elution was performed at 70 °C using pure water at a flow rate of 1 mL/min. The concentrations of eluted compounds were calculated by internal standard methods [30]. The citrate concentration was determined by HPLC using column SH1011 (Shodex. Japan), eluent was 5 mM HCLO_4_, flow rate 1 mL/min, detector was VIS at 430 nm.

The mass yield of erythritol (*Y*_*ERY*_) was expressed in *g/g* from glucose and was calculated from the equation *Y*_*ERY*_ = *P/S*. The volumetric productivity (*Q*_*ERY*_) was expressed in g/L h and was calculated from *Q*_*ERY*_=* P/V·t*, where P is the amount of erythritol in the culture liquid at the end of fermentation (g); S is the total amount of glucose consumed (g); *V* is the initial volume of culture liquid (L), and *t* is the culture time (h). Glucose consumption rate (r_GLU_) was calculated as the amount of glucose consumed per hour and per liter of culture medium.

### General molecular biological techniques

Standard media and techniques were used for *E. coli* [31], and the media and techniques used for *Y. lipolytica* have been described elsewhere [32]. The restriction enzymes, DNA polymerases, and ligase were supplied by Thermo Fisher Scientific. Genomic DNA from *Y. lipolytica* was prepared in accordance with Cheng et al. [33]. PCR was performed using the primers listed in Additional file 1: Table S1. Dream Taq DNA polymerase (Thermo Scientific) was used for cloning, and ExTaq DNA polymerase (Takara) was used to verify the gene integration in the genome. The PCR fragments were purified from the agarose gels using a GeneJet Gel Extraction Kit (Thermo Scientific). DNA sequencing and primers synthesis were performed by Sangon Biotech (https://www.sangon.com). Yeast transformation was as described in An et al. [34].

### Sequence analysis

Protein BLAST searches were performed on National Center for Biotechnology Information web server (http://blast.ncbi.nlm.nih.gov/Blast). The phylogenetic tree of putative *ER* genes was constructed with MEGA7 software [35] using the neighbor-joining method [36]. Bootstrap analysis was used with 1000 replicates to test the relative support for the branches produced by the neighbor-joining analysis. The evolutionary distances were computed using the JTT matrix-based method [37]. The rate variation among sites was modeled with a gamma distribution (shape parameter of 1). Evolutionary analyses were performed based on the alignment of the full-length amino acids sequences. All the analyzed sequences of aldose reductase enzymes were retrieved from GenBank and SWISS-PROT databases.

### Cloning of ER encoding genes

Putative ER genes (Table 1) were PCR amplified for genomic DNA of strain CGMCC7326 using primer listed in Additional file 1: Table S1. Forward (F) and reverse (R) primers (name according to gene nomenclature) were designed to introduce a *Nde*I and *Xho*I site in the resulting amplicons, respectively. Amplicons were digested by *Nde*I and *Xho*I and cloned into pET28a expression vector at the corresponding sites. Strain *E. coli* BL21(DE3) was then transformed with the different constructs. The correctness of the resulting vectors was verified by DNA sequencing using a T7 primer (Additional file 1: Table S1).

### Disruption of ER encoding genes

To construct the ER10 disruption cassette, 2 kb DNA fragment located upstream (P) and downstream (T) of gene g141.t1 were PCR amplified using genomic DNA of strain CGMCC7326 as a template, and primers pairs ER10-F1/ER10-R1 and ER10-F2/ER10-R2, respectively. After purification, P fragment was digested with *Not*I and *Sal*I and cloned at corresponding sites of plasmid pINA-Pir1-A.oryFTase [38], to yield plasmid pINA-UP_ER10_ (Additional file 1: Table S1). The purified T fragment was then digested with *Eco*RI and *Not*I, and cloned at corresponding sites of plasmid pINA-UP_ER10_, to yield plasmid pINA-UP_ER10_-DW_ER10_. Similarly, to construct ER25 disruption cassette, P and T fragments of gene g3023.t1 were amplified using primer pairs ER25-F1/ER25-R1 and ER25-F2/ER25-R2, respectively. Purified P and T fragments were then digested with *Not*I-*Sal*I and *Eco*RI-*Not*I, respectively, and cloned in two steps in plasmid pHyg-Pir1-PdSIase [39] to yield final plasmid pINA-UP_ER25_-DW_ER25_. The ER27 gene (g801.t1) disruption plasmid pINA-UP_ER27_-DW_ER27_ was constructed similarly with primers pairs ER27-F1/ER27-R1 and ER27-F2/ER27-R2. P and T fragments were digested with *Not*I-*Sal*I and *Eco*RI-*Not*I, respectively, and cloned in two steps in plasmid pHP4d-Pir1-A.oryGal [34]. The above plasmids were then *Not*I digested and the purified ER disruption cassettes were used to transform *Y. lipolytica* strain CGMCC7326. Transformants were selected using sucrose, lactose or hygromycin B as a selectable marker. The correctness of genes disruption in the resulting strains HCY100, HCY101, and HCY102 was verified by analytical PCR using primer pair ER10-F/ER10-R, ER25-F/ER25-R and ER27-F/ER27-R (Additional file 1: Table S1). To construct strain disrupted for ER10, ER25 and ER27, the disruption cassettes released from plasmid pINA-UP_ER10_-DW_ER10_ and pINA-UP_ER27_-DW_ER27_ after *Not*I digestion were used to transform sequentially strain HCY101. After selection of transformants on medium containing sucrose (YNBS) and lactose (YNBL), the correctness of ER10 and ER27 gene disruption was verified by analytical PCR using primer pairs ER10-F/ER10-R and ER27-F/ER27-R, respectively. The strain deleted for the three ER encoding genes was named as HCY103 (Additional file 1: Table S1).

### Overexpression of ER encoding genes

Genes g141.t1 (ER10), g3023.t1 (ER25) and g801.t1 (ER27) were amplified with primer pairs ER10-F11/ER10-R11, ER25-F11/ER25-R11, and ER27-F11/ER27-R11, respectively, and genomic DNA of strain CGMCC7326 as a template. Primers F and R were designed to introduce a *Hin*dIII and *Bam*HI site at, respectively, 5′ and 3′ end of each ER encoding gene (Additional file 1: Table S1). Purified amplicons were then digested using *Hind*III and *Bam*HI, and cloned at the corresponding sites of plasmid pHyg-Pir1-PdSIase [39], a derivative of plasmid pINA1313 [40] that allow random integrations into the yeast genome. The resulting constructs were named pINA-ER10, pINA-ER25, and pINA-ER27, respectively (Additional file 1: Table S1). The correctness of the resulting constructs was verified by DNA sequencing. Expression cassettes for genes g141.t1 (ER10), g3023.t1 (ER25) and g801.t1 (ER27) were rescued from the corresponding vectors by *Not*I digestion. They were then purified from the agarose gel and used to transform *Y. lipolytica* strain CGMCC7326. Transformants were selected on YPD medium supplemented with hygromycin B (200 μg/mL). The resulting strains were named HCY104 (php4d-ER10), HCY105 (php4d-ER25), HCY106 (php4d-ER27). To construct strain HCY107 (Additional file 1: Table S1), that overexpress the three ER genes (ER10 + ER25 + ER27), the hygromycin concentration was increased to allow additional integration of the ER overexpression cassette.

We started from a strain overexpressing *ER10* (HCY104) and we transformed it with *ER25* expression cassette. We then selected the corresponding transformant (genotype *ER10*-*ER25*) on medium containing 800 μg/mL of hygromycin. This strain was then transformed with *ER27* expression cassette and transformants were selected and 2000 μg/mL of hygromycin. In brief, transformants were selected on YPD supplemented with hydromycin B at a concentration of 200 μg/mL of hygromycin for mono-integration, 800 μg/mL for double-integration and 2000 μg/mL for triple-integration.

### Overexpression of gene *ZWF1* and *GND1*

Genes *ZWF1* (*YALI0E22649g*) and *GND1* (*YALI0B15598g*) from *Y. lipolytica* were also overexpressed in the strain HCY107. A DNA fragment containing genes *ZWF1* and *GND1* under the control of the constitutive *hp8d* promoter and *XDH* gene from *Scheffersomyces stipitis* CBS 6054 used as a selectable marker was synthesized by GENEWIZ (Suzhou, China), and cloned at *Eco*RI site of pUC57 to yield pUC57-zwf-gnd (see Additional file 1: Figure S6 for details). The *ZWF1* and *GND* expression cassette rescued from pUC57-zwf-gnd by using *EcoR*I were then used to transform strain HCY107. Transformants were selected using xylitol as a selectable marker (YNBX). The final strain that overexpresses genes *ER10*, *ER25*, *ER27*, *ZWF1* and *GND1* was designated as HCY108.

### RNA isolation and transcript quantification

Shake-flask cultures were grown in EPMG medium. Cells were collected at an OD_600_ of 2.0 and stored at − 80 °C in Trizol solution. Total RNAs were extracted using the TRNzol kit from TianGen Biotech (Beijing, China), and cDNA was obtained using PrimeScript™ RT reagent Kit with gDNA Eraser (Takara, Dalian, China). The qRT-PCRs were performed using SYBR^®^ Premix ExTaq™ II (TliRNaseH Plus), Takara, Dalian, China) and ABI7500 Real-Time PCR system (Applied Biosystems). Primer pairs for qRT-PCRs were, ER10-F_fluo_/ER10-R_fluo_, ER25-F_fluo_/ER25-R_fluo_, ER27-F_fluo_/ER27-R_fluo_ for gene ER10, ER25 and ER27, respectively (Additional file 1: Table S1). Gene expressions were normalized to that of the actin gene (primers β-actin-up/β-actin-down; ∆CT method). The fold differences in ER gene expression between the transformants and the control strains CGMCC7326 were calculated as 2^−ΔΔCT^ [41]. All samples were analyzed in triplicate.

### Protein production and purification

A single colony of recombinant *E. coli* BL21 (DE3) was grown in 5 mL of Luria–Bertani medium (LB) with 100 μg/mL kanamycin in 30-mL universal tubes at 37 °C for 3 h with shaking at 220 rpm. Thereafter, the 5 mL of cells were transferred to 495 mL of LB medium with 100 μg/mL kanamycin in a 2-L Erlenmeyer flask. The cells were incubated at 37 °C with shaking at 220 rpm until the OD_600_ reached 1.0. Then, isopropyl-*β*-d-thiogalactopyranoside (IPTG) was added at a final concentration of 1 mM and cultures were further incubated at 25 °C for 10 h (until OD_600_ reached 4.5). Two hundred and fifty milliliters of cells were harvested by centrifugation at 12,000*g* and 4 °C for 10 min, before being resuspended in 100 mL binding buffer (20 mM Tris–HCl pH 8.0, 200 mM NaCl, 1 mM PMSF, and 2 mM *β*-mercaptoethanol). Cells were sonicated at 4 °C for twenty cycles of 5 s at 5 s interval. Cell debris was removed by centrifugation at 10,000*g* for 10 min at 4 °C. The cell extract was finally filtered through a 0.45 μm membrane filter (Millipore).

Erythrose reductase from cell lysates was purified using Ni–nitrilotriacetate agarose (Ni–NTA) according to the manufacturer recommendation. Proteins were eluted with a buffer containing 25 mM NaH_2_PO_4_-150 mM NaCl-200 mM imidazole (pH 8.0). The Ni–NTA purified erythrose reductases were further applied to gel filtration on Sephadex G-25 and eluted with 50 mM Tris–Cl, 200 mM NaCl, pH 8.0 following the manufacturer’s instructions. SDS-PAGE was performed in a 12% (w/v) acrylamide gel at a constant current of 200 mA. Low molecular weight protein standard was used as size marker (GenScript, NJ, USA). After electrophoresis, gels were stained with Coomassie brilliant blue G-250 via standard procedures. Scanning densitometry of SDS-PAGE gel was performed using Phoretics 1D Non-linear Dynamics (Newcastle, UK) software.

### Enzyme assay

The accurate assays of erythrose reductase activity or its *Km* values were problematic as commercial d-erythrose (Sigma Catalog No. E7625-250 mg) tested were only 75% purity. In order to evaluate more accurately its activity and *Km* values, we purified it by semi-preparative HPLC to obtain d-erythrose with 95% purity.

Erythrose reductase activity was determined by monitoring the reduction or oxidation of cofactor NADP(H) by absorbance measurement at 340 nm [5, 7]. The assay mixture (1.5 mL) contained 50 mM phosphate buffer (pH 6), 2 mM of co-factor (NADP or NADPH), 10 mM of substrate unless stated otherwise (erythrose or erythritol), and 0.2 mL of enzyme solution (cell extract or purified ER). This reaction mixture was allowed to stand for one min to eliminate any endogenous oxidation of cofactor before adding the substrate. The arabitol and mannitol dehydrogenase activity were tested according to the method described by Cheng et al. [42]. One unit of enzyme activity corresponds to 1 μmol of cofactor consumed or generated per minute at 30 °C. Activities were expressed as units/mg of protein and the results correspond to mean value of triplicate assays. The value used for ε of NADPH and NAD was as 6.22 mM/cm.

### Kinetic parameters

The kinetic parameters of the purified ER enzymes were calculated. For this purpose, initial velocity studies were performed with erythrose as a variable substrate concentration ranged from 2 to 200 mM in the presence of a fixed concentration of cofactor (NADPH, 2 mM). When using erythritol as a substrate, the concentration varied from 20 to 1500 mM in the presence of NADP^+^ (2 mM). Kinetic parameters were calculated by non-linear regression of velocities against substrate concentrations, and processed using OriginPro 8.0 software.

### Effect of pH and metal ions on ER enzyme relative activity

The influence of pH on the activity of ERs was carried out at 30 °C in the Mcilvaine’s buffer (from pH 3.0 to 8.0), containing 10 mM erythrose and 2 mM NADPH and 50 μL of purified ER enzymes (1 mg/mL). The assays were performed in triplicate at each pH point. The effect of metal ions on ER activity was studied by assaying different ZnSO_4_, CuSO_4_, MnSO_4_, NiSO_4_ and CaCl_2_ concentrations (from 0.1 to 5 mM) at 30 °C in 25 mM Mcilvaine’s buffer (pH 6.0) using erythrose as a substrate.

### Substrate specificity of ER enzymes

The substrate specificity assays were performed using erythritol, d-erythrose, d-fructose, d-glucose, d-galactose, d-arabinose, l-arabinose, xylose, ribose, xylulose as substrate. The reaction mixture contained (1.5 mL final volume) 50 mM substrate, 2 mM NADPH, 50 μL of purified ER enzymes and 25 mM potassium phosphate buffer (pH 6.0). Specificity was expressed as U/mg of protein and normalized to that obtained for d-erythrose. For cofactor specificity, ER activity on erythrose (10 mM) was determined as described above using cofactor (NADH or NADPH) at a concentration of 2 mM and 10 mM. Oxidation of erythritol was measured in the same conditions in the presence of NADP or NAD.

## Additional file

**Additional file 1: Table S1.** Primers, gene cassettes, and strains used in this study. **Table S2.** Identity of YALI0D07634p (ER10), YALI0C13508p (ER25), YALI0F18590p (ER27) with ER from *Candida magnoliae* ER (ACT78580.1), *Trichosporonoides megachiliensis* ER1 (BAD90687), *Tilletiaria anomala* ER3 (XP_013243550.1) and *Moniliella* sp. ER3 (AGB07593.1). **Table S3.** Effect of divalent metal ions on the activities of ER10 and ER25. **Figure S1.** SDS-PAGE analysis of the ER10, ER25, and ER27 overexpressed in *E. coli* BL21(DE3). Lane M: protein standards; lane 1: crude extract of non-induced cells; lane 2: crude extract of IPTG-induced cells; lane 3: purified ER protein by Ni^2+^ affinity resin; lane 4: purified ER protein by gel-filtration. **Figure S2.** The non-linear regression plots of initial-velocity against D-erythrose (A, C, D) or erythritol (B) using ER10 (A, B), ER25 (C) and ER27 (D) as enzymes. **Figure S3.** Amino acid sequences of ER27, ER25, and ER10. The specific motif IPKSXXXXR is highlighted in bold. **Figure S4.** Comparison of HPLC spectrum of culture supernatant of the *Y. lipolytica* wild-type strain CGMCC7326 and engineered strains, namely HCY104 (php4d-ER10), HCY105 (php4d-ER25), HCY106 (php4d-ER27), HCY108 *php4d-ER10*-25-27, p*hp8d-ZWF1*-*GND1*). **Figure S5.** pH or OD_600_ change during fermentation of strains CGMCC7326, HCY104, HCY107, HCY108, at the starting pH 6.5 (A, C), and pH 3.0 (B, D). For the control strain CGMCC7326, pH was decreased to 3.2 in 48 h and retained around 2.9±0.2 during fermentation until the depletion of glucose, and pH increased to 3.4 after glucose was completely exhausted, when the starting pH was 6.5 (A); For the engineered strains (HCY104, HCY107, and HCY108), pH was decreased to 4.6±0.05 in 24 h and maintained around 4.2±0.2 during fermentation until the depletion of glucose, and pH increased to 4.6±0.1 after glucose was depleted, when the starting pH was 6.5 (A); For the control strain CGMCC7326, the pH was maintained around 2.9±0.1 when fermented at starting pH 3 buffered with citrate until glucose was depleted (B). For the engineered strain, the pH was maintained around 3.2±0.2 when fermented at starting pH 3 buffered with citrate until glucose was depleted (B). The control strain produced more cell biomass and citrate than the engineered strains whenever at starting pH 6.5 or 3.0 (C, D). **Figure S6.** Schematic representation of the DNA fragment used to overexpress *ZWF1* and *GND1* genes in strain HCY108.
