# Supplementary material for: Identification, characterization of two NADPH-dependent erythrose reductases in the yeast Yarrowia lipolytica and improvement of erythritol productivity using metabolic engineering
Source: Microb Cell Fact. 2018 Aug 29;17:133. doi: 10.1186/s12934-018-0982-z (PMC6114734; doi:10.1186/s12934-018-0982-z)
Supplement: Supplementary file 1 — Additional file 1: Table S1. Primers, gene cassettes, and strains used in this study. Table S2. Identity of YALI0D07634p (ER10), YALI0C13508p (ER25), YALI0F18590p (ER27) with ER from Candida magnoliae ER (ACT78580.1), Trichosporonoides megachiliensis ER1 (BAD90687), Tilletiaria anomala ER3 (XP_013243550.1) and Moniliella sp. ER3 (AGB07593.1). Table S3. Effect of divalent metal ions on the activities of ER10 and ER25. Figure S1. SDS-PAGE analysis of the ER10, ER25, and ER27 overexpressed in E. coli BL21(DE3). Lane M: protein standards; lane 1: crude extract of non-induced cells; lane 2: crude extract of IPTG-induced cells; lane 3: purified ER protein by Ni2+ affinity resin; lane 4: purified ER protein by gel-filtration. Figure S2. The non-linear regression plots of initial-velocity against D-erythrose (A, C, D) or erythritol (B) using ER10 (A, B), ER25 (C) and ER27 (D) as enzymes. Figure S3. Amino acid sequences of ER27, ER25, and ER10. The specific motif IPKSXXXXR is highlighted in bold. Figure S4. Comparison of HPLC spectrum of culture supernatant of the Y. lipolytica wild-type strain CGMCC7326 and engineered strains, namely HCY104 (php4d-ER10), HCY105 (php4d-ER25), HCY106 (php4d-ER27), HCY108 php4d-ER10-25-27, php8d-ZWF1-GND1). Figure S5. pH or OD600 change during fermentation of strains CGMCC7326, HCY104, HCY107, HCY108, at the starting pH 6.5 (A, C), and pH 3.0 (B, D). For the control strain CGMCC7326, pH was decreased to 3.2 in 48 h and retained around 2.9±0.2 during fermentation until the depletion of glucose, and pH increased to 3.4 after glucose was completely exhausted, when the starting pH was 6.5 (A); For the engineered strains (HCY104, HCY107, and HCY108), pH was decreased to 4.6±0.05 in 24 h and maintained around 4.2±0.2 during fermentation until the depletion of glucose, and pH increased to 4.6±0.1 after glucose was depleted, when the starting pH was 6.5 (A); For the control strain CGMCC7326, the pH was maintained around 2.9±0.1 when fermented at start [file 12934_2018_982_MOESM1_ESM.docx]

**Identification, Characterization of Two NADPH-Dependent Erythrose Reductases in the Yeast *Yarrowia lipolytica* And Improvement of Erythritol productivity Using Metabolic Engineering**

Huiling Cheng^a*^, Siqi Wang^a*^, Muhammad Bilal^a^, Xuemei Ge^b^, Can Zhang^c^, Patrick Fickers^d^, Hairong Cheng^a^

^a^State Key Laboratory of Microbial Metabolism, and School of Life Sciences & Biotechnology, Shanghai Jiao Tong University, Shanghai, China;

^b^College of Light Industry and Food Engineering, Nanjing Forestry University, Nanjing, China;

^c^School of Pharmacy, Shanghai Jiao Tong University, Shanghai, China;

^d^Microbial Processes and Interactions, TERRA Teaching and Research Centre, University of Liège - Gembloux Agro-Bio Tech, Belgium

^*^These authors contributed equally to this study.

**Additional materials**

**Additional Tables**

| **Table S1** Primers, gene cassettes, and strains used in this study | | |
| --- | --- | --- |
| Strains/Plasmids/  Primers | Genotype/Sequences (5'→3') | References/  Restriction sites |
| ***E. coli strain*** |  |  |
| BL21(DE3) | B F^–^ *ompT* *gal* *dcm* *lon* *hsdS_B_*(*r_B_*^–^*m_B_*^–^) λ(DE3 [*lacI* *lacUV5*-*T7p07* *ind1* *sam7* *nin5*]) [*malB*^+^]_K-12_(λ^S^) pLysS[*T7p20* *ori*_p15A_](Cm^R^) | New England BioLabs |
| TOP10 | F– *mcr*AΔ(*mrr*-*hsd*RMS-*mcr*BC) Φ80*lac*ZΔM15 Δ*lac*X74 *rec*A1 *ara*D139 Δ(*ara leu*) 7697 *gal*U *gal*K *rps*L (StrR) *end*A1 *nup*G | Thermo Fisher Scientific |
| HCE100 | BL21(DE3) pET28a-ER5 | This work |
| HCE101 | BL21(DE3) pET28a-ER8 | This work |
| HCE102 | BL21(DE3) pET28a-ER10 | This work |
| HCE103 | BL21(DE3) pET28a-ER14 | This work |
| HCE104 | BL21(DE3) pET28a-ER16 | This work |
| HCE105 | BL21(DE3) pET28a-ER17 | This work |
| HCE106 | BL21(DE3) pET28a-ER18 | This work |
| HCE107 | BL21(DE3) pET28a-ER20 | This work |
| HCE108 | BL21(DE3) pET28a-ER22 | This work |
| HCE109 | BL21(DE3) pET28a-ER24 | This work |
| HCE110 | BL21(DE3) pET28a-ER25 | This work |
| HCE111 | BL21(DE3) pET28a-ER27 | This work |
| ***Y. lipolytica* strains** | Genotype |  |
| CGMCC7326 | *Suc-*, *Lac-*, *Mal-* | 14 |
| HCY100 | CGMCC7326 derivative, *ER10::A.oryFTase* | This work |
| HCY101 | CGMCC7326 derivative, *ER25::PdSIase* | This work |
| HCY102 | CGMCC7326 derivative, *ER27::A.oryGal* | This work |
| HCY103 | CGMCC7326 derivative, *ER10::A.oryFTase, ER25::PdSIase,, ER27::A.oryGal* | This work |
| HCY104 | CGMCC7326 derivative, php4d-*ER10* | This work |
| HCY105 | CGMCC7326 derivative, php4d-*ER25* | This work |
| HCY106 | CGMCC7326 derivative, php4d-*ER27* | This work |
| HCY107 | CGMCC7326 derivative, php4d-*ER10,* php4d-*ER25,* php4d-*ER27* | This work |
| HCY108 | HCY107derivative, php8d-*ZWF1*-*GND1* | This work |
| **Plasmids** |  |  |
| pUC57 | LacZ, AmpR | Thermo Fischer Scientific |
| pET28A | LacI, T7 promoter, KanR | Novagen |
| pET28a-ER5 | pET28a derivative containing gene g413.t1 (*YALI0F09075g*) | This work |
| pET28a-ER8 | pET28a derivative containing gene g414.t1(*YALI0F09097g*) | This work |
| pET28a-ER10 | pET28a derivative containing gene g141.t1 (*YALI0D07634g*) | This work |
| pET28a-ER14 | pET28a derivative containing gene g3584.t1 (*YALI0C02805g*) | This work |
| pET28a-ER16 | pET28a derivative containing gene g5767.t1 (*YALI0B07117g*) | This work |
| pET28a-ER17 | pET28a derivative containing gene g973.t1 (*YALI0A15906g*) | This work |
| pET28a-ER18 | pET28a derivative containing gene g5171.t1 (*YALI0B15268g*) | This work |
| pET28a-ER20 | pET28a derivative containing gene g3251.t1 (*YALI0C20251g*) | This work |
| pET28a-ER22 | pET28a derivative containing gene g3449.t1 (*YALI0C06171g*) | This work |
| pET28a-ER24 | pET28a derivative containing gene g5456.t1 (*YALI0B01298g*) | This work |
| pET28a-ER25 | pET28a derivative containing gene g3023.t1 (*YALI0C13508g*) | This work |
| pET28a-ER27 | pET28a derivative containing gene g801.t1(*YALI0F18590g*) | This work |
| pINA-UP_ER10_-DW_ER10_ | *UP_ER10_-ura3d1-hp4d-Pir1-A.oryFTase-LIP2t-DW_ER10_* | This work |
| pINA-UP_ER25_-DW_ER25_ | *UP_ER25_-hyg-hp4d-hp4d-Pir1-PdSIase-LIP2t-DW_ER25_* | This work |
| pINA-UP_ER27_-DW_ER27_ | *UP_ER27_-ura3d1-hp4d-Pir1-A.oryGal-LIP2t-DW_ER27_* | This work |
| pINA-ER10 | *'zeta-hyg-hp4d-hp4d-ER10-LIP2t-zeta'* | This work |
| pINA-ER25 | *'zeta-hyg-hp4d-hp4d-ER25-LIP2t-zeta'* | This work |
| pINA-ER27 | *'zeta-hyg-hp4d-hp4d-ER27-LIP2t-zeta'* | This work |
| **Primers** |  |  |
| P g413.t1-F | ATACATATGTCTGTAC TGCTCACCGG ATCCAC | *Nde*I |
| P g413.t1-R | ATACTCGAGTTACAACTTG TTGATATACT TGGGATC | *Xho*I |
| Pg414.t1-R | ATACTCGAGTTACTCCTCC TTCTTAACTT CCAAC | *Xho*I |
| Pg414.t1-F | ATACATATGTCCATTC TTGTCACCGG CGCCAC | *Nde*I |
| Pg141.t1-F | ATACATATGTCCTTCA AGCTCGCCTC CGGAAAG | *Nde*I |
| Pg141.t1-R | ATACTCGAGTTAGGCGAAA ATGGGAAGGT TAGCG | *Xho*I |
| Pg3584.t1-F | ATACATATGGCCAAGA ACCAGGAACT TTTCC | *Nde*I |
| Pg3584.t1-R | ATACTCGAGTTACAGAGCA GCACCCAGAT GGGCAG | *Xho*I |
| Pg5767.t1-F | ATACATATGTTCCGGT CAGTATATAA ACGGG | *Nde*I |
| Pg5767.t1-R | ATACTCGAGCTAGCAGAAG TCAAAGTCGG GGAATC | *Xho*I |
| Pg973.t1-F | ATACATATGACTTCCA TCGACTTTAC CATGAAC | *Nde*I |
| Pg973.t1-R | ATACTCGAGTTAGCAAAAG TCAAAGTCGG GGAAAC | *Xho*I |
| Pg5171.t1-F | ATACATATGACTGTGC CCAAAGTGAA ACTATC | *Nde*I |
| Pg5171.t1-R | ATACTCGAGTTATATTTTG GCTTCGCCCT CTTCGG | *Xho*I |
| Pg3251.t1-F | ATACATATGCCCACTA CACTCGTCAC TGGAGC | *Nde*I |
| Pg3251.t1-R | ATACTCGAGTTACTTATGC TGCTCGTACC ACTGG | *Xho*I |
| Pg3449.t1-F | ATACATATGTCCTACA CCCGAGTCAC TCTTC | *Nde*I |
| Pg3449.t1-R | ATACTCGAGTTACAGCTTG CTGTCCAGCT TGTAG | *Xho*I |
| Pg5456.t1-F | ATACATATGACTACCA CTGCCACAGA GACCC | *Nde*I |
| Pg5456.t1-R | ATACTCGAGTTAGTTGAAG AGTCTCGACC AAAATC | *Xho*I |
| Pg3023.t1-F | ATACATATGCCAATTA TCACAGAAAC ATTCAAG | *Nde*I |
| Pg3023.t1-R | ATACTCGAGTTATTTGTCC TCGAAACCAA GGTTG | *Xho*I |
| Pg801.t1-F | ATACATATGGCAGGCG GACCCACTCT CAAGC | *Nde*I |
| Pg801.t1-R | ATACTCGAGTTACTTCTTC TGCTCAGCAA GGTAC | *Xho*I |
| ER10-F1 | AATGCGGCCGCCGTGAGCGACCGCCGGAGCG ACCTATATG | *Not*I |
| ER10-R1 | AATGTCGACGTTCAATTGG TGTGTTTGGG TTGATAGG | *Sal*I |
| ER10-F2 | AATGAATTCCACGGAAACGACTGGCCAAA GGTTAGAAGT | *Eco*RI |
| ER10-R2 | AA GCGGCCGCGTTCGGTACACGACCCTCGCCTGTCTG | *Not*I |
| ER25-F1 | AATGCGGCCGCTGCTGCTCCCCCGTTCTCAAACTCCT | *Not*I |
| ER25-R1 | AATGTCGACGGTGTGCGTGGGTACAAGTGCAGT | *Sal*I |
| ER25-F2 | AAT GAATTCGCGACTTCAGTGACCTGATCGAAT | *Eco*RI |
| ER25-R2 | AATGCGGCCGCGGTAACCGACGTAACCGGCGGA | *Not*I |
| ER27-F1 | AATGCGGCCGCGGGCCTTTCGCATTTGCTGCCCCA | *Not*I |
| ER27-R1 | AATGTCGACCTGTGAATGTCTTCTGGGTTGGTTGT | *Sal*I |
| ER27-F2 | AATGAATTCGTACCCAGCGGTATCAGTTGAGA | *Eco*RI |
| ER27-R2 | AATGCGGCCGCATTGCGCCCAGATTGCGCCTTGAGA | *Not*I |
| ER10-F | ATGTCCTTCA AGCTCGCCTC CGGAAAG |  |
| ER10-R | TTA GGC GAA TTA GGG AAG GTT AG |  |
| ER25-F | ATGCCAATTATCACAGAAACATTCAAG C |  |
| ER25-R | CTATTTGTCCTCGAAACCAAGGTTGTCGC |  |
| ER27-F | ATGGCAGGCGGACCCACTCTCAAGCTC |  |
| ER27-R | TTACTTCTTCTGCTCAGCAAGGTACTTC |  |
| ER10-F11 | AATAAGCTTATGTCCTTCA AGCTCGCCTC CGGAAAG | *Hin*dIII |
| ER10-R11 | AATGGATTCTTA GGC GAA TTA GGG AAG GTT AG | *Bam*HI |
| ER25-F11 | AATAAGCTTATGCCAATTATCACAGAAACATTCAAG C | *Hin*dIII |
| ER25-R11 | AATGGATTCCTATTTGTCCTCGAAACCAAGGTTGTCGC | *Bam*HI |
| ER27-F11 | AATAAGCTTATGGCAGGCGGACCCACTCTCAAGCTC | *Hin*dIII |
| ER27-R11 | AATGGATTCTTACTTCTTCTGCTCAGCAAGGTACTTC | *Bam*HI |
| ER10-F*_fluo_* | GAAGGAGTGGGCTGGATGGA |  |
| ER10-R*_fluo_* | GTTCTGGGCGGTGAAGTTGG |  |
| ER25-F*_fluo_* | GCCCACTACACCGACAACAAT |  |
| ER25-R*_fluo_* | GTGACCAGAATGCCGTGCTT |  |
| ER27-F*_fluo_* | TGCTGCCCTCATTGTCAACTG |  |
| ER27-R*_fluo_* | ACTCCTCGTCAAGGCTGTCCA |  |
| T7 | TAATACGACTCACTATAGGGAGA |  |
| *β*-actin-up | ACTCTGGTGAT GTGTCACCCACG |  |
| *β*-actin-down | TCGGACGATTTCTCGCTCGGCGGAG |  |
| Note: Underlined nucleotides indicate restriction sites used for cloning. *hp4d* are synthetic promoters, *TT_xpr2_* and *TT_ura3_* are terminators. | | |

| **Table S2**. Identity of YALI0D07634p (ER10), YALI0C13508p (ER25), YALI0F18590p (ER27) with ER from *Candida magnoliae* ER (ACT78580.1), *Trichosporonoides megachiliensis* ER1 (BAD90687), *Tilletiaria anomala* ER3 (XP_013243550.1) and *Moniliella* sp. ER3 (AGB07593.1) | | | |  |
| --- | --- | --- | --- | --- |
| Protein ID | YALI0D07634p  (ER10) | YALI0C13508p  (ER25) | YALI0F18590p  (ER27) | |
| YALI0D07634p (ER10) | 100 | 26.8 | 31.0 | |
| YALI0C13508p (ER25) | 26.8 | 100 | 31.8 | |
| YALI0F18590p (ER27) | 31.0 | 31.8 | 100 | |
| BAD90687 | 29.9 | 29.9 | 37.4 | |
| ACT78580.1 | 24.0 | 25.7 | 31.3 | |
| AGB07593.1 | 29.6 | 29.0 | 37.1 | |
| XP_013243550.1 | 27.4 | 29.9 | 38.0 | |

| **Table S3** Effect of divalent metal ions on the activities of ER10 and ER25 | | | | | | | | | | |
| --- | --- | --- | --- | --- | --- | --- | --- | --- | --- | --- |
| Conc.  (mM) | Relative activity (%) | | | | | | | | | |
|  | ZnSO_4_ | | CuSO_4_ | | MnCl_2_ | | NiSO_4_ | | CaCl_2_ | |
|  | ER10 | ER25 | ER10 | ER25 | ER10 | ER25 | ER10 | ER25 | ER10 | ER25 |
| 0 | 100 | 100 | 100 | 100 | 100 | 100 | 100 | 100 | 100 | 100 |
| 0.5 | 111 | 106 | 103 | 98 | 80 | 93 | 72 | 129 | 90 | 95 |
| 1 | 109 | 111 | 85 | 97 | 74 | 90 | 70 | 123 | 88 | 90 |
| 5 | 31 | 6 | 70 | 36 | 71 | 25 | 52 | 95 | 66 | 84 |
| The values provided are the means of two independent replicates; the standard deviations represented less than 10% of the means. Specific activity obtained without the addition of divalent metal ions was fixed at 100%. | | | | | | | | | | |

**Additional Figures**


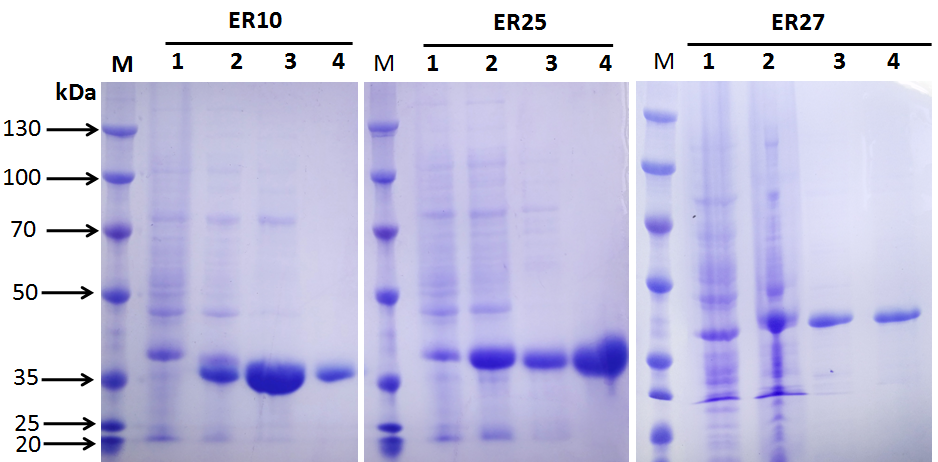


**Figure S1.** SDS-PAGE analysis of the ER10, ER25, and ER27 overexpressed in *E. coli* BL21(DE3). Lane M: protein standards; lane 1: crude extract of non-induced cells; lane 2: crude extract of IPTG-induced cells; lane 3: purified ER protein by Ni^2+^ affinity resin; lane 4: purified ER protein by gel-filtration.


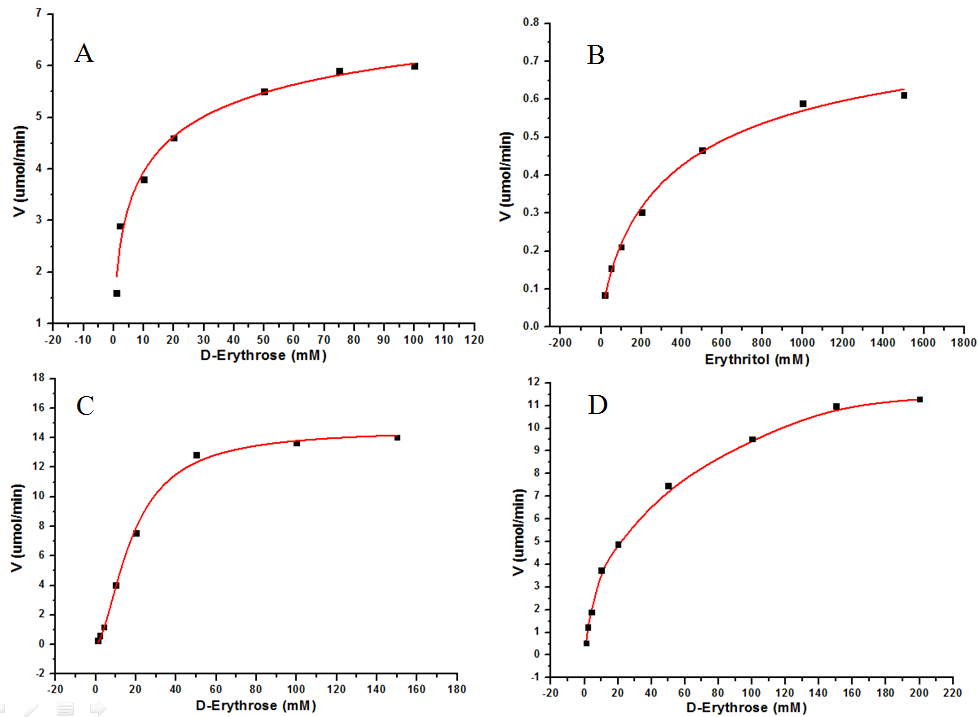


**Figure S2.** The non-linear regression plots of initial-velocity against D-erythrose (A, C, D) or erythritol (B) using ER10 (A, B), ER25 (C) and ER27 (D) as enzymes.

**
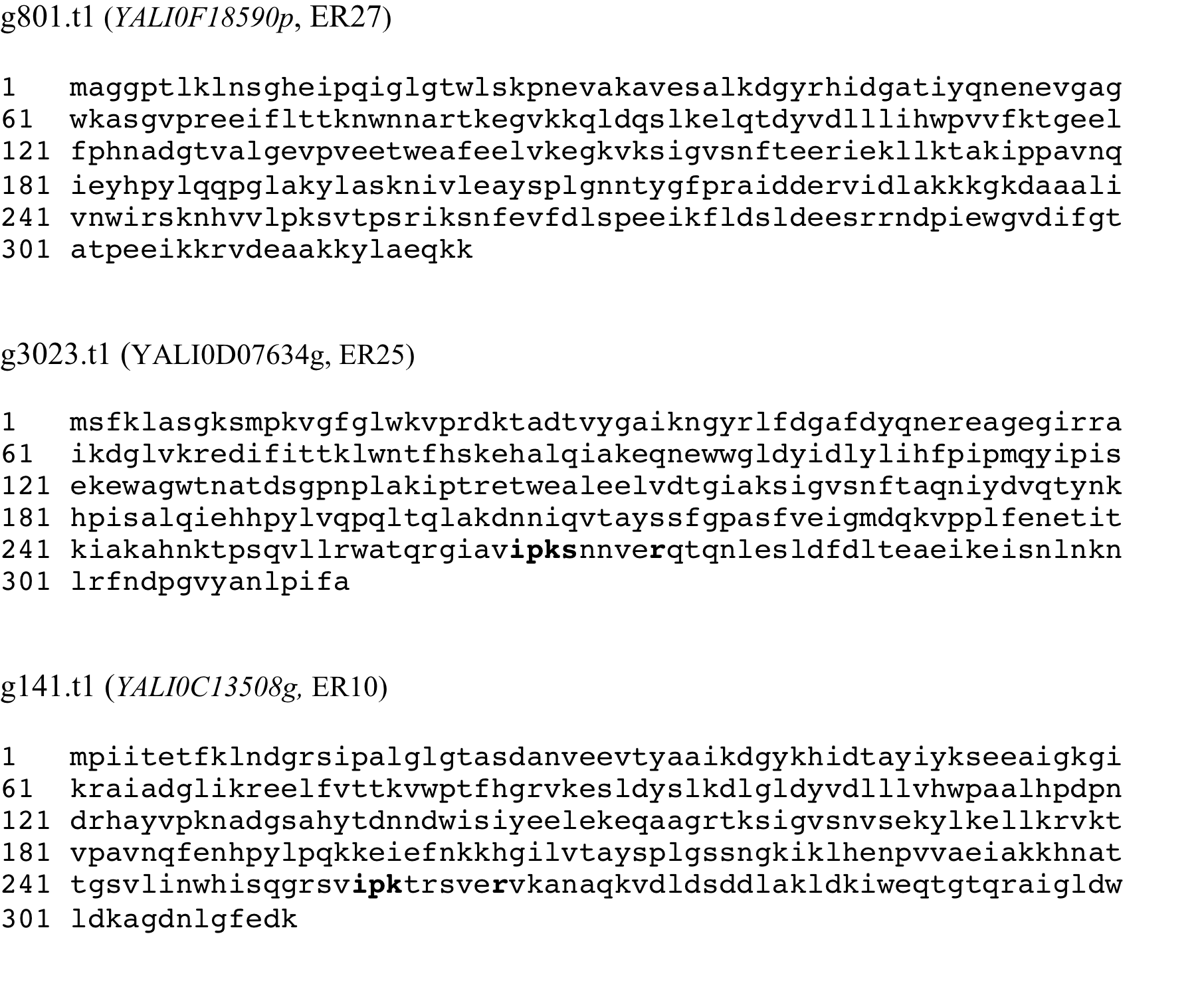
**

**Figure S3.** Amino acid sequences of ER27, ER25, and ER10. The specific motif IPKSXXXXR is highlighted in bold.


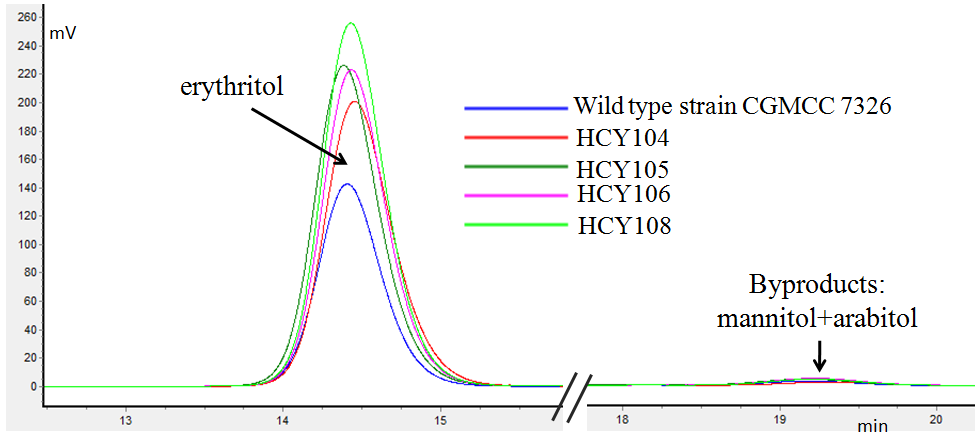


**Figure S4.** Comparison of HPLC spectrum of culture supernatant of the *Y. lipolytica* wild-type strain CGMCC7326 and engineered strains, namely HCY104 (php4d-ER10), HCY105 (php4d-ER25), HCY106 (php4d-ER27), HCY108 *php4d-ER10*-25-27, p*hp8d-ZWF1*-*GND1*).


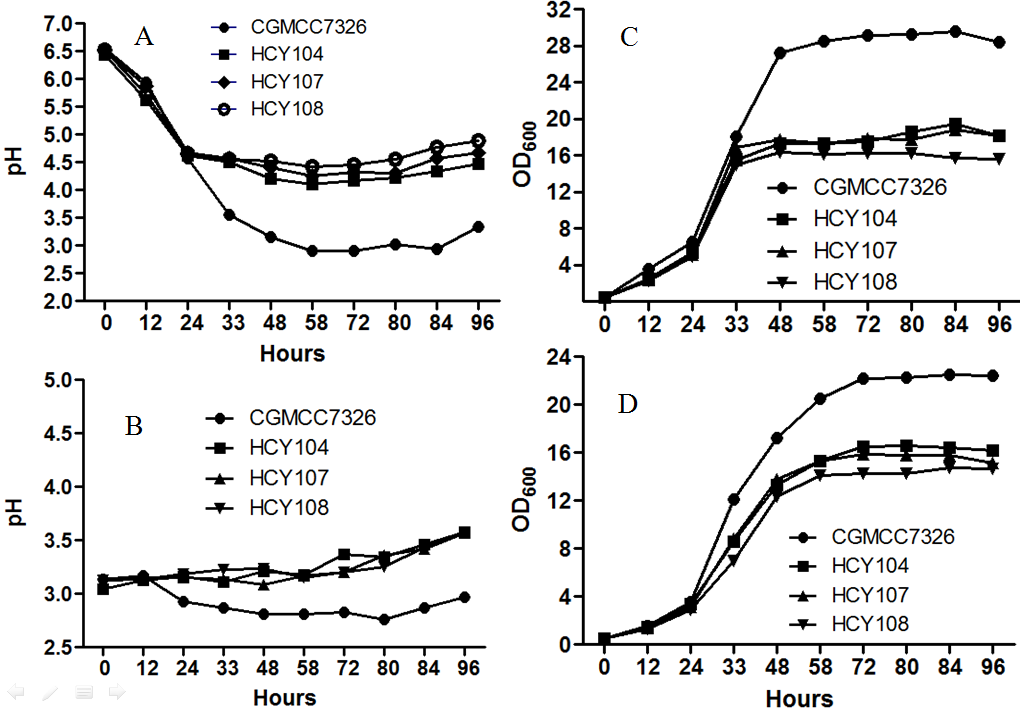


**Figure S5.** pH or OD_600_ change during fermentation of strains CGMCC7326, HCY104, HCY107, HCY108, at the starting pH 6.5 (A, C), and pH 3.0 (B, D).

For the control strain CGMCC7326, pH was decreased to 3.2 in 48 h and retained around 2.9±0.2 during fermentation until the depletion of glucose, and pH increased to 3.4 after glucose was completely exhausted, when the starting pH was 6.5 (A); For the engineered strains (HCY104, HCY107, and HCY108), pH was decreased to 4.6±0.05 in 24 h and maintained around 4.2±0.2 during fermentation until the depletion of glucose, and pH increased to 4.6±0.1 after glucose was depleted, when the starting pH was 6.5 (A); For the control strain CGMCC7326, the pH was maintained around 2.9±0.1 when fermented at starting pH 3 buffered with citrate until glucose was depleted (B). For the engineered strain, the pH was maintained around 3.2±0.2 when fermented at starting pH 3 buffered with citrate until glucose was depleted (B). The control strain produced more cell biomass and citrate than the engineered strains whenever at starting pH 6.5 or 3.0 (C, D).


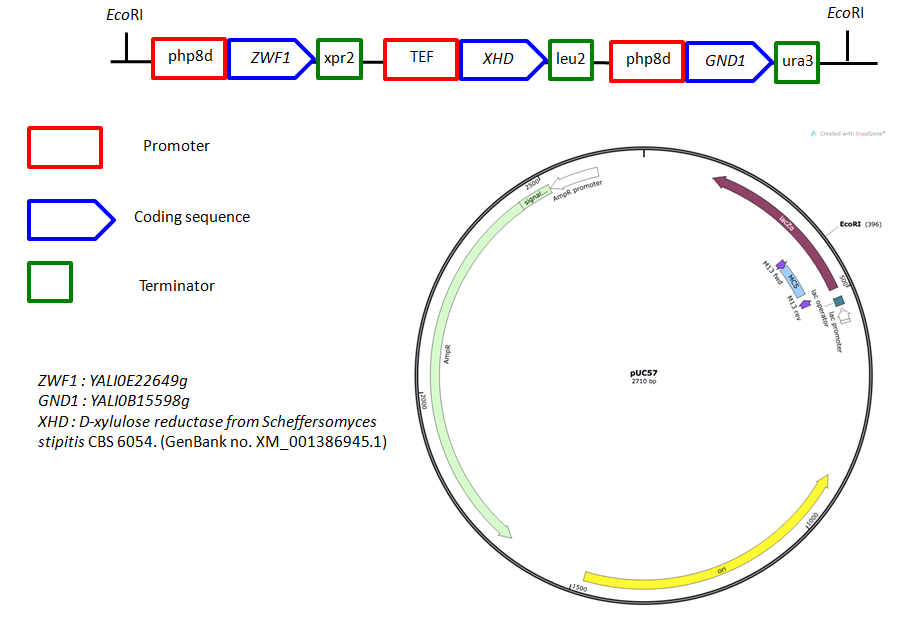


**Figure S6.** Schematic representation of the DNA fragment used to overexpress *ZWF1* and *GND1* genes in strain HCY108.
